# Stochastic simulations of the tetracycline operon

**DOI:** 10.1186/1752-0509-5-9

**Published:** 2011-01-19

**Authors:** Konstantinos Biliouris, Prodromos Daoutidis, Yiannis N Kaznessis

**Affiliations:** 1 Department of Chemical Engineering and Materials Science, University of Minnesota, 421 Washington Ave SE, Minneapolis, MN 55455 USA

## Abstract

**Background:**

The tetracycline operon is a self-regulated system. It is found naturally in bacteria where it confers resistance to antibiotic tetracycline. Because of the performance of the molecular elements of the tetracycline operon, these elements are widely used as parts of synthetic gene networks where the protein production can be efficiently turned on and off in response to the presence or the absence of tetracycline. In this paper, we investigate the dynamics of the tetracycline operon. To this end, we develop a mathematical model guided by experimental findings. Our model consists of biochemical reactions that capture the biomolecular interactions of this intriguing system. Having in mind that small biological systems are subjects to stochasticity, we use a stochastic algorithm to simulate the tetracycline operon behavior. A sensitivity analysis of two critical parameters embodied this system is also performed providing a useful understanding of the function of this system.

**Results:**

Simulations generate a timeline of biomolecular events that confer resistance to bacteria against tetracycline. We monitor the amounts of intracellular TetR2 and TetA proteins, the two important regulatory and resistance molecules, as a function of intrecellular tetracycline. We find that lack of one of the promoters of the tetracycline operon has no influence on the total behavior of this system inferring that this promoter is not essential for *Escherichia coli*. Sensitivity analysis with respect to the binding strength of tetracycline to repressor and of repressor to operators suggests that these two parameters play a predominant role in the behavior of the system. The results of the simulations agree well with experimental observations such as tight repression, fast gene expression, induction with tetracycline, and small intracellular TetR2 amounts.

**Conclusions:**

Computer simulations of the tetracycline operon afford augmented insight into the interplay between its molecular components. They provide useful explanations of how the components and their interactions have evolved to best serve bacteria carrying this operon. Therefore, simulations may assist in designing novel gene network architectures consisting of tetracycline operon components.

## Background

Recent advances in our ability to mathematically investigate the dynamic complexity of biomolecular systems, have created inroads into these systems. Examples include natural systems, such as the lactose [1,2] and tryptophan [3] operon, and synthetic systems such as the oscillator [4,5], logic AND gates [6] and toggle switch [7]. In the present paper, we examine the dynamic behavior of the tetracycline (*tet*) operon. Although some studies have examined the interactions of different parts of the *tet *operon [8-10], to our knowledge there is no mathematical model that describes all the biomolecular interactions of this intriguing system. The *tet *operon is found naturally in bacteria where it confers resistance to antibiotic tetracycline (Tc).

Tc used to be one of the most common antibiotics for treating bacterial infections. It functions by binding the bacterial ribosome (Rib), thereby impeding the process of translation (protein biosynthesis) and causing cell death [11]. Due to its low cost, Tc was used excessively. Because of the excessive use, bacteria developed resistance to it.

**Figure 1:**
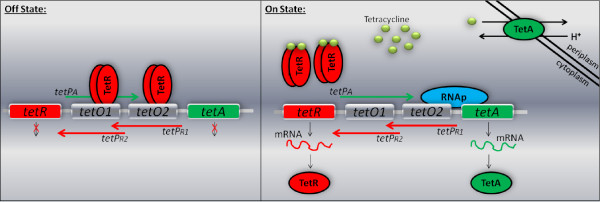
**Schematic representation of the tet operon without (off state) and with (on state) Tc**. When Tc is absent, the cells save energy by repressing the expression of *tetA *and *tetR*. When Tc is present, TetA is produced and pumps Tc out of the cell. More TetR is also produced to shut down expression again when it is no longer necessary [16].

Already in 1964, there was evidence that *Escherichia coli *(*E. coli*) bacteria had developed resistance to Tc, but the exact resistance mechanism was not clear [12]. To date, four resistance mechanisms have been identified [13]. These mechanisms are associated with a) active efflux of Tc out of the cell, b) Rib protection from Tc, c) rRNA mutation and d) Tc inactivation. In the present work, we investigate the mechanism of active transport of Tc out of the cell, whereby bacteria under attack by Tc, quickly produce a membrane protein that pumps Tc out of the cell. This resistance mechanism relies on the *tet *operon [14]. Several Tc resistance determinants have evolved [13,15]. This work refers to class B (or Tn10-type) Tc resistance determinant. A qualitative model of the *tet *operon is shown in Figure 1.

The *tet *operon comprises of two genes, *tetA *and *tetR*. The first encodes TetA, a transporter protein which removes Tc from the cell. The *tetR *gene encodes TetR which binds to the operators, acts as a repressor and inhibits the expression of both genes in the absence of Tc. Moreover, this operon consists of three promoters and two operators. One promoter belongs to the *tetA *gene while the other two belong to the *tetR *gene. The two operators control the expression of the two genes. The promoters and the operators overlap, significantly increasing the complexity of this biological switch.

In the absence of Tc, TetR binds as a dimer (TetR2) to both operators. TetR2 binding to the operators results in the repression of gene expression. When Tc enters the cell, it binds to Rib preventing protein synthesis. Importantly, it also binds to the TetR2 repressor, with high affinity, causing a conformational change in the DNA binding region. This results in TetR2 unbinding from the operator sites. Once TetR2 has dissociated from the operator sites, gene expression is turned on producing TetA and TetR. Subsequently, Tc is removed from the cell through the active transport mechanism mediated by TetA. After the expulsion of Tc from the cell, TetR2 protein binds the operator sites again, turning off the expression of the two genes [11,14,16-21].

It is interesting to note that expression of genes is activated only in the presence of the antibiotic Tc. This is a remarkable cost-effective mechanism, a feature that has made the molecular components (promoters, operators, repressors) of this system attractive for the design and development of a gamut of synthetic gene networks [4,6,8,22-24]. The molecular elements of this operon are widely used as parts of biological switches, taking advantage of the fast and robust switching. More specifically, the expression of desired proteins is placed under the transcriptional control of the *tet *components, where protein production can be efficiently turned on and off in response to the presence or the absence of Tc.

Herein, we formulate a mathematical model of the naturally occurring *tet *operon based on experimental findings. Our model incorporates all the biomolecular interactions of this system including those involved in dimerization, transcription, translation, degradation, repression and induction. The model represents each elementary biomolecular interaction with biochemical reactions.

We use a hybrid, stochastic-discrete and stochastic-continuous algorithm to simulate the behavior of the *tet *operon in order to understand how it responds to various disturbances [25,26]. The use of the stochastic algorithm allows us to consider single cell time lines as well as variability across different cells. Lastly, a sensitivity analysis of the binding strength of Tc for TetR2 and of TetR2 for the operator sites is performed, providing mechanistic insight into the functionality of this system.

## Methods

### Model description

How to best model biological systems is still an open question. Here we adhere to Monod's postulate that biological complexity can be reduced to networks of biomolecular interactions [27]. Our model consists of 40 species and 61 biochemical reactions which capture the biomolecular interactions occurring in the *tet *operon. The entire reaction network is supplied in Table 1 and can be found online at http://www.synbioss.org/. SynBioSS is an open source software suite for modeling biological networks [28-30].

**Table 1:** Reaction network of the *tet *operon.

Number	Reaction
1	$2TetR\overset{k_{1}}{\underset{k_{2}}{⇌}}TetR2$
2	$TetR2+tetO1\overset{k_{3}}{\underset{k_{4}}{⇌}}TetR2:tetO1$
3	$TetR2+tetO2\overset{k_{5}}{\underset{k_{6}}{⇌}}TetR2:tetO2$
4	$TetR2+nsDNA\overset{k_{7}}{\underset{k_{8}}{⇌}}TetR2:nsDNA$

5	$TcEx\overset{k_{9}}{\to }Tc$

6	$TetR2+Tc\overset{k_{10}}{\underset{k_{11}}{⇌}}TetR2:Tc$
7	$TetR2:Tc+Tc\overset{k_{12}}{\underset{k_{13}}{⇌}}TetR2:Tc2$
8	$TetR2:tetO1+Tc\overset{k_{14}}{\underset{k_{15}}{⇌}}TetR2:tetO1:Tc$
9	$TetR2:tetO1:Tc+Tc\overset{k_{16}}{\underset{k_{17}}{⇌}}TetR2:tetO1:Tc2$
10	$TetR2:tetO1:Tc2\overset{k_{18}}{\underset{k_{19}}{⇌}}TetR2:Tc2+tetO1$
11	$TetR2:tetO2+Tc\overset{k_{20}}{\underset{k_{21}}{⇌}}TetR2:tetO2:Tc$
12	$TetR2:tetO2:Tc+Tc\overset{k_{22}}{\underset{k_{23}}{⇌}}TetR2:tetO2:Tc2$
13	$TetR2:tetO2:Tc2\overset{k_{24}}{\underset{k_{25}}{⇌}}TetR2:Tc2+tetO2$

14	$RN\;Ap+Complex\overset{k_{26}}{\underset{k_{27}}{⇌}}RN\;Ap:\left[tetP_{R1}\right]:tetP_{R2}:tetP_{A}:tetO1:tetO2$
15	$RN\;Ap:\left[tetP_{R1}\right]:tetP_{R2}:tetP_{A}:tetO1:tetO2\overset{k_{28}}{\to }RN\;Ap*:\left[tetP_{R1}\right]:tetP_{R2}:tetP_{A}:tetO1:tetO2$
16	$RN\;Ap*:\left[tetP_{R1}\right]:tetP_{R2}:tetP_{A}:tetO1:tetO2\overset{k_{29}}{\to }RN\;Ap*:DN\;A\left(P_{R1}\right)+Complex$
17	$RN\;Ap*:DN\;A\left(P_{R1}\right)\overset{k_{30}}{\underset{k_{31}}{\to }}RN\;Ap+mRN\;A\left(tetR\right)$

18	$RN\;Ap+Complex\overset{k_{32}}{\underset{k_{33}}{⇌}}RN\;Ap:tetP_{R1}:\left[tetP_{R2}\right]:tetP_{A}:tetO1:tetO2$
19	$RN\;Ap:tetP_{R1}:\left[tetP_{R2}\right]:tetP_{A}:tetO1:tetO2\overset{k_{34}}{\to }RN\;Ap*:tetP_{R1}:\left[tetP_{R2}\right]:tetP_{A}:tetO1:tetO2$
20	$RN\;Ap*:tetP_{R1}:\left[tetP_{R2}\right]:tetP_{A}:tetO1:tetO2\overset{k_{35}}{\to }RN\;Ap*:DN\;A\left(P_{R2}\right)+Complex$
21	$RN\;Ap*:DN\;A\left(P_{R2}\right)\overset{k_{36}}{\underset{k_{37}}{\to }}RN\;Ap+mRN\;A\left(tetR\right)$

22	$RN\;Ap+tetP_{R2}+tetO1+TetR2:tetO2\overset{k_{38}}{\underset{k_{39}}{⇌}}RN\;Ap:\left[tetP_{R2}\right]:tetO1:TetR2:tetO2$
23	$RN\;Ap:\left[tetP_{R2}\right]:tetO1:TetR2:tetO2\overset{k_{40}}{\to }RN\;Ap*:\left[tetP_{R2}\right]:tetO1:TetR2:tetO2$
24	$RN\;Ap*:\left[tetP_{R2}\right]:tetO1:TetR2:tetO2\overset{k_{41}}{\to }RN\;Ap*:DN\;A\left(P_{R2}\right)+tetP_{R2}+tetO1:TetR2:tetO2$

25	$Rib+mRN\;A\left(tetR\right)\overset{k_{42}}{\to }Rib:mRN\;A\left(tetR\right)$
26	$Rib:mRN\;A\left(tetR\right)\overset{k_{43}}{\to }Rib*:mRN\;A\left(tetR\right)+mRN\;A\left(tetR\right)$
27	$Rib*:mRN\;A\left(tetR\right)\overset{k_{44}}{\underset{k_{45}}{\to }}Rib+tetR$

28	$RN\;Ap+Complex\overset{k_{46}}{\underset{k_{47}}{⇌}}RN\;Ap:tetP_{R1}:tetP_{R2}:\left[tetP_{A}\right]:tetO1:tetO2$
29	$RN\;Ap:tetP_{R1}:tetP_{R2}:\left[tetP_{A}\right]:tetO1:tetO2\overset{k_{48}}{\to }RN\;Ap*:tetP_{R1}:tetP_{R2}:\left[tetP_{A}\right]:tetO1:tetO2$
30	$RN\;Ap*:tetP_{R1}:tetP_{R2}:[tetP_{A}]:tetO1:tetO2\overset{k_{49}}{\to }RN\;Ap*:DN\;A\left(P_{A}\right)+Complex$
31	$RN\;Ap*:DN\;A\left(P_{A}\right)\overset{k_{50}}{\underset{k_{51}}{\to }}RN\;Ap+mRN\;A\left(tetA\right)$
32	$Rib+mRN\;A\left(tetA\right)\overset{k_{52}}{\to }Rib:mRN\;A\left(tetA\right)$
33	$Rib:mRN\;A\left(tetA\right)\overset{k_{53}}{\to }Rib*:mRN\;A\left(tetA\right)+mRN\;A\left(tetA\right)$
34	$Rib*:mRN\;A\left(tetA\right)\overset{k_{54}}{\underset{k_{55}}{\to }}Rib+TetA$

35	TetR2→k560
36	$TetR2:Tc2\overset{k_{57}}{\to }2Tc$
37	mRN A(tetR)→k580
38	mRN A(tetA)→k590
39	Tc→k600

40	$Tc+TetA\overset{k_{61}}{\underset{k_{62}}{\to }}TetA$

41	$tetP_{R1}+tetP_{R2}+tetP_{A}+tetO1+tetO2\overset{k_{63}}{\to }Complex$
42	$Complex+TetR2\overset{k_{64}}{\to }TetR2:tetO1+tetP_{R1}+tetP_{R2}+tetP_{A}+tetO2$
43	$Complex+TetR2\overset{k_{65}}{\to }TetR2:tetO2+tetP_{R1}+tetP_{R2}+tetP_{A}+tetO1$
44	$Complex+TetR2:Tc2\overset{k_{66}}{\to }TetR2:tetO1:Tc2+tetP_{R1}+tetP_{R2}+tetP_{A}+tetO2$
45	$Complex+TetR2:Tc2\overset{k_{67}}{\to }TetR2:tetO2:Tc2+tetP_{R1}+tetP_{R2}+tetP_{A}+tetO1$

The definition of the species that participate in the *tet *operon reaction network and the kinetic constants of the reactions are found in Table 2 and Table 3 respectively. In what follows, we provide a description of the salient features of this operon. We explain also the reactions that we use to describe the biomolecular processes taking place in this system.

**Table 2:** Definition of the species that participate in the *tet *operon reaction network.

Species	Definition
TcEx	External Tc
Tc	Intracellular Tc
TetR	Tc repressor protein
TetR2	Tc repressor protein (dimer)
TetA	Tc transport protein
*tetO1*	Operator site 1
*tetO2*	Operator site 2
nsDNA	Non-specific DNA sites
*tetP*_*R*1_	Promoter 1 of *tetR *gene
*tetP*_*R*2_	Promoter 2 of *tetR *gene
*tetP_A_*	Promoter of *tetA *gene
RNAp	RNA polymerase
Complex	Free operator and promoter sites
mRNA(*tetA*)	*tetA *gene mRNA
mRNA(*tetR*)	*tetR *gene mRNA
Rib	Ribosome
*Rib*:*mRNA*(*tetR*)	Ribosome bound to mRNA(*tetR*)
*Rib*:*mRNA*(*tetA*)	Ribosome bound to mRNA(*tetA*)
*TetR*2:*tetO1*	TetR2 bound to *tetO1*
*TetR*2:*tetO2*	TetR2 bound to *tetO2*
*TetR*2:*nsDNA*	TetR2 bound to nsDNA
*TetR*2:*Tc*	TetR2 bound to one Tc molecule
*TetR*2:*Tc*2	TetR2 bound to two Tc molecules
*TetR*2:*tetO1*:*Tc*	TetR2 bound to *tetO1 *and one Tc molecule
*TetR*2:*tetO1*:*Tc*2	TetR2 bound to *tetO1 *and two Tc molecules
*TetR*2:*tetO2*:*Tc*	TetR2 bound to *tetO2 *and one Tc molecule
*TetR*2:*tetO2*:*Tc*2	TetR2 bound to *tetO2 *and two Tc molecules
*RNAp*:[*tetP*_*R*1_]:*tetP*_*R*2_:*tetP_A_*:*tetO1*:*tetO2*	Closed complex of RNAp bound to *tetP*_*R*1_
RNAp*:[*tetP*_*R*1_]:*tetP*_*R*2 _:*tetP_A_*:*tetO1*:*tetO2*	Open complex of RNAp bound to *tetP*_*R*1_
*RNAp*:*tetP*_*R*1 _:[*tetP*_*R*2_]:*tetPA*:*tetO1*:*tetO2*	Closed complex of RNAp bound to *tetP*_*R*2_
RNAp*:*tetP*_*R*1 _:[*tetP*_*R*2_]:*tetP_A_*:*tetO1*:*tetO2*	Open complex of RNAp bound to *tetP*_*R*2_
*RNAp*:[*tetP*_*R*2_]:*tetO1*:*TetR*2:*tetO2*	Closed complex of RNAp bound to *tetP*_*R*2_
RNAp*:[*tetP*_*R*2_]:*tetO1*:*tet*_*R*2_:*tetO2*	Open complex of RNAp bound to *tetP*_*R*2_
*RNAp*:*tetP*_*R*1 _:*tetP*_*R*2 _:[*tetP_A_*]:*tetO1*:*tetO2*	Closed complex of RNAp bound to *tetP_A_*
RNAp*:*tetP*_*R*1 _:*tetP*_*R*2 _:[*tetP_A_*]:*tetO1*:*tetO2*	Open complex of RNAp bound to *tetP_A_*
Rib*:*mRNA*(*tetA*)	Rib bound to the first codon of the mRNA(*tetA*)
Rib*:*mRNA*(*tetR*)	Rib bound to the first codon of the mRNA(*tetR*)
RNAp*:*DNA*(*P_A_*)	RNAp bound to *tetA *gene
RNAp*:*DNA*(*P*_*R*1_)	RNAp bound to *tetR *gene starting transcription from *tetP*_*R*1_
RNAp*:*DNA*(*P*_*R*2_)	RNAp bound to *tetR *gene starting transcription from *tetP*_*R*2_

**Table 3:** Kinetic constants of the *tet *operon reaction network.

kinetic constant	**Ref**.	kinetic constant	**Ref**.	kinetic constant	**Ref**.	kinetic constant	**Ref**.
*k*_1 _= 10^+9^	[31]^h^	*k*_18 _= 5.80·10^-3^	[31]	*k*_35 _= 30	[44]	*k*_52 _= 10^+5^	e
*k*_2 _= 10	[31]^h^	*k*_19 _= 0.10	[20]	*k*_36 _= 30	[44]^a^	*k*_53 _= 100	[44]
*k*_3 _= 10^+8^	[31]^h^	*k*_20 _= 4.80·10^+5^	[20]	*k*_37 _= 621	[8]^b^	*k*_54 _= 100	[44]^a^
*k*_4 _= 10^-4^	[31]^h^	*k*_21 _= 10^-4^	[20]	*k*_38 _= 7.23·10^+6^	[40,63]^h^	*k*_55 _= 394	[64]^b^
*k*_5 _= 5.00·10^+8^	[16,31]^h^	*k*_22 _= 2.40·10^+5^	[20]	*k*_39 _= 0.10	[63]^h^	*k*_56 _= 3.85·10^-5^	f
*k*_6 _= 10^-4^	[31]^h^	*k*_23 _= 2.00·10^-4^	[20]	*k*_40 _= 0.013	[63]	*k*_57 _= 3.85·10^-5^	f
*k*_7 _= 30	[31]^h^	*k*_24 _= 5.80·10^-3^	[31]	*k*_41 _= 30	[44]	*k*_58 _= 0.002	e
*k*_8 _= 0.10	[31]^h^	*k*_25 _= 0.10	[20]	*k*_42 _= 10^+5^	e	*k*_59 _= 0.002	e
*k*_9 _= 3.30·10^-4^	[65]	*k*_26 _= 4.8·10^+4^	[40,63]^h^	*k*_43 _= 100	[44]	*k*_60 _= 2.67*·*10^-6^	[43]
*k*_10 _= 4.80·10^+5^	[20]	*k*_27 _= 0.10	[63]^h^	*k*_44 _= 100	[44]^a^	*k*_61 _= 1	[13]^c^
*k*_11 _= 10^-4^	[20]	*k*_28 _= 0.013	[63]	*k*_45 _= 207	[8]^b^	*k*_62 _= 4.90·10^-5^	[21]^d^
*k*_12 _= 2.40·10^+5^	[20]	*k*_29 _= 30	[44]	*k*_46 _= 8.60·10^+6^	[19,63]^h^	*k*_63 _= 10^+8^	g
*k*_13 _= 2.00·10^-4^	[20]	*k*_30 _= 30	[44]^a^	*k*_47 _= 0.10	[63]^h^	*k*_64 _= 10^+8^	[31]
*k*_14 _= 4.80·10^+5^	[20]	*k*_31 _= 621	[8]^b^	*k*_48 _= 0.013	[63]	*k*_65 _= 5.00·10^+8^	[16,31]
*k*_15 _= 10^-4^	[20]	*k*_32 _= 9.10·10^+5^	[40,63]^h^	*k*_49 _= 30	[44]	*k*_66 _= 0.10	[20]
*k*_16 _= 2.40·10^+5^	[20]	*k*_33 _= 0.10	[63]^h^	*k*_50 _= 30	[44]^a^	*k*_67 _= 0.10	[20]
*k*_17 _= 2.00·10^-4^	[20]	*k*_34 _= 0.013	[63]	*k*_51 _= 1182	[64]^b^		

Units on k: first order reactions: *sec*^-1^, second order reactions: *M*^-1^*sec*^-1^, third order reactions: *M*^-2^*sec*^-1^, fourth order reactions: *M*^-3^*sec*^-1^, fifth order reactions: *M*^-4^*sec*^-1^.

^a ^Scale parameter of the gamma distributed event (*sec*).

^b ^Shape parameter of the gamma distributed event.

^c ^*k_cat _*of Michaelis Menten kinetics.

^d ^*K_M _*of Michaelis Menten kinetics.

^e ^Rate adjusted to give 20 protein molecules per mRNA transcript.

^f ^Rate adjusted for 5 hours half life.

^g ^Rate adjusted for very fast reaction.

^h ^Rate calculated through equilibrium constant by assuming the forward and calculating the reverse rate.

As illustrated in Figure 1, the *tet *operon is composed of 2 genes, *tetR *and *tetA*. These give rise to two proteins, TetR and TetA. Let us start with the interactions of TetR. TetR2, the dimer form of TetR, is involved in inhibiting gene expression in the absence of Tc [31]. We model the dimerization of the TetR as a second order reversible reaction (reaction 1).

TetR2 protein represses the system by binding to the operator sites of the *tet *operon. We designate these two operators as *tetO1 *and *tetO2*. The binding of the repressor protein to the *tetO1 *shuts down the expression of both the two genes. On the other hand, the binding to the *tetO2 *impedes the expression of the *tetA *gene and only down regulates the expression of the *tetR *gene [32]. There is no indication of cooperativity between these two operator sites [33]. We represent the binding of TetR2 to the operator sites, *tetO1 *and *tetO2*, as a second order bimolecular reaction (reactions 2,3).

TetR2 can bind to non-specific DNA (nsDNA) sites as well [33]. Even though the affinity of the repressor protein for the nsDNA is low, there are about 4 million nsDNA sites in the *E. coli *genome thereby significantly increasing the probability of TetR2 being bound to nsDNA. These non-specific binding interactions are captured by the reaction 4.

It is noteworthy that the affinity of the repressor for *tetO2 *is about 5 times higher than for *tetO1 *[34]. It has been theorized that this mechanism is used by *E. coli *to avoid fortuitous expression of the *tetA *gene. An unexpected decrease in the number of TetR protein would favor the repression of the *tetA *gene first, until more TetR proteins are produced and expression of the two genes is inhibited again. In any case, the repressor shuts down the expression of the *tetA *gene first and soonafter the expression of the *tetR *gene [16].

The diffusion of the Tc into the cell is represented by a first order reaction capturing the effective rate at which Tc enters the cell (reaction 5). We should note that although Tc is certain to bind other targets in the cell, in our model we only regard an effective Tc amount that interacts only with the *tet *operon. Once Tc diffuses into the cell, it binds to a metal ion, M, yielding the complex M-Tc [20]. For simplicity, in our model, we neglect the formation of this complex which occurs almost instantaneously. Subsequently, Tc binds to the repressor (TetR2 bound to the operators or free) with high affinity. Upon binding, TetR2 undergoes a conformational change that reduces dramatically its affinity for the operators [35,36]. The Tc binding to TetR2 is coded as a second order bimolecular reaction. Each TetR2 protein harbors two binding sites. The two Tc molecules bind in succession, and without cooperativity, to yield TetR2:Tc2 [20,37]. These interactions are described by reactions 6-13.

When the operator sites are free from TetR2, transcription will occur. Transcription can be separated into three stages: initiation, elongation and termination. In order for transcription initiation to occur, the holoenzyme, which is composed of sigma factor and RNA polymerase (RNAp), is recruited to the promoter sites. There it forms a closed complex [38]. This entire process is effectively modeled as a second order bimolecular reaction (reaction 14). After the formation of the closed complex, the DNA double helix is unwound forming an open complex (reaction 15). Once the open complex has been formed, RNAp starts to transcribe each single nucleotide and the sigma factor is released from the holoenzyme. The transcription of the first nucleotide is considered a first order reaction (reaction 16). Subsequently, RNAp moves along the DNA transcribing each nucleotide independently of the others [38]. This process could be modeled as a series of reactions, each of them representing the transcription of each single nucleotide. Considering the reaction times as exponentially distributed events, we integrate all these reactions in a single reaction that represents a gamma distributed event (reaction 17) [39]. The scale parameter is equal to the rate of the transcription of each single nucleotide while the shape parameter equals the number of nucleotides that are transcribed.

As shown in Figure 1, there are three promoter binding sites in the *tet *operon where the holoenzyme is recruited. We designate these binding sites as *tetP*_*R*1_, *tetP*_*R*2 _and *tetP_A_*. *tetP*_*R*1 _and *tetP*_*R*2 _regulate the *tetR *gene whereas *tetP_A _*controls the *tetA *gene. If an RNAp is recruited to one of the three sites, it prohibits another RNAp from being recruited to one of the other two [18]. Furthermore, the affinity of RNAp for each of the three sites is different [19,40]. Thus, we include three different cases (one case for each single promoter) to capture all the different possible ways that transcription initiation can occur. We model the first case (site *tetP*_*R*1_) using the reactions 14-17.

Reactions 14-17 depict the transcription of the *tetR *gene when an RNAp is recruited to the *tetP*_*R*1 _site. The *tetP*_*R*1 _site accounts for approximately 5% of the total mRNA transcripts of the *tetR *gene [40]. In Table 1, the brackets indicate the promoter site from which transcription initiation occurs. In reaction 14, *Complex *refers to the DNA molecule when all of the promoter sites and the operators are free. When all the sites are free, they form a single, contiguous complex that RNAp can bind to and start transcription. The formation of this complex is described in reaction 41. It should be stressed that this reaction has no physical meaning and it serves as an algorithmic trick. To this end, reactions 42-45 are the same as forward reactions 2 and 3 and backward reactions 10 and 13. Similarly to reactions 14-17, reactions 18-21 represent the transcription of *tetR *gene when RNAp binds to the promoter site *tetP*_*R*2_ (second case).

*Tet *operon is a tightly regulated system. One of its striking features is that even if the repressor (TetR2 protein) is bound to the operator *tetO2*, RNAp can still be recruited to the promoter *tetP*_*R*2 _and start transcription if *tetO1 *is free [32]. The affinity of RNAp for the *tetP*_*R*2 _is slightly lower in this case than in the case when both the promoters, *tetO1 *and *tetO2*, are free. This mechanism is modeled by reactions 22-24.

Similarly to the process of transcription, the process of translation also progresses in three steps: initiation, elongation, and termination. The initiation stage includes the association of mRNA with the ribosomal units (50S and 30S) and the initiator tRNA to form the initiation complex [38]. This entire process is represented as a first order irreversible reaction (reaction 25). After the initiation complex forms, Rib translates the mRNA molecule into its protein products [38]. The movement of the Rib from the ribosome binding site to the coding region along with the release of the mRNA molecule are described in reaction 26. The elongation process is integrated, as in the transcription process, in one gamma distributed reaction event (reaction 27) [39]. In this case the scale parameter is equal to the rate of the translation of each single codon and the shape parameter equals the number of amino acids that are produced. The species utilized in transcription and translation (Rib, RNAp, promoters, operators) are finally freed to participate in these processes again. The translation mechanisms are shown in reactions 25-27.

We use the same pattern of reactions to model the expression of the *tetA *gene as we did to model *tetR *expression. In this case, there is only one promoter site, namely *tetP_A_*, where transcription initiation can take place (third case). Using a reaction formalism to capture transcription and translation we come up with reactions 28-34.

The proteins as well as the mRNAs are degraded during the cell life. mRNAs are degraded by ribonuclease enzymes. The protein degradation is catalyzed by proteases [41]. The degradation of both the mRNA and proteins is modeled as simple first order reactions (reactions 35-38). Reactions 35 and 36 refer to the degradation of TetR2 protein. TetR2 can be degraded when it is free (reaction 35) or when it is bound by Tc (reaction 36). To our knowledge, there is no study that demonstrates TetA degradation. Therefore, we assume that TetA is not degraded, a plausible assumption for any membrane protein. However, in our model TetA gets diluted due to cell division. Further, we consider Tc degradation (reaction 39).

Finally, reaction 40 captures the removal of Tc from the cell. As mentioned above, the exclusion of Tc from the cell occurs through a process controlled by the TetA protein. This process is energy dependent and is driven by a pH gradient across the membrane [21]. The removal of one Tc molecule is coupled by the influx of one proton in the cell [17]. Eventually, TetA remains in the membrane of the cell while Tc is pumped out of the cell. There is evidence that TetA protein can exist as a dimer or even as a multimer in the cell, but this is still unclear [42]. For the sake of simplicity, in our formulation we consider TetA to exist as a monomer.

### Model parameters

In Table 3, we present the values of the parameters for the model. The *tet *operon is one of the best studied bacterial gene networks. As such, there is an atypical wealth of parameters on the strength of biomolecular interactions. Therefore, most of the equilibrium constants that we used in our model have been obtained through experimental procedures. The relevant literature references are presented in Table 3.

A concern may be legitimately posed that in most cases strengths of biomolecular interactions are measured *in vitro*, that is outside of the pertinent biological context. Indeed, this is a quintessential challenge faced by quantitative biology. This is a reason we chose to study the sensitivity of the operon behavior to changes in the values of important parameters.

In most cases of the reversible reactions, only the equilibrium constant is actually available. Then the kinetic constant of the forward reaction is assumed and the reverse reaction kinetic constant is subsequently calculated through the equilibrium constant.

Having in mind that the TetR dimerization equilibrium constant is greater than 10^7^*M*^-1 ^[31], we consider it equal to 10^8^*M*^-1 ^(equal to the one observed for the lactose repressor, LacI) [8]. Regarding reaction 41, we set a reaction rate equal to 10^8 ^aiming to make it a very fast reaction.

As regards the binding of Tc to TetR2, it was modeled based on [37]. More specifically, the kinetic constant of the binding of the first Tc molecule to TetR2 (either free or bound to operators) is twice the kinetic constant of the binding of the second Tc molecule to TetR2. Consequently, the kinetic constant of the unbinding of the second Tc molecule from TetR2 is twice the kinetic constant of the unbinding of the first Tc molecule.

Another choice we made was on the degradation rate of TetR2, which is chosen with 5 hours half time. The rate at which Tc is degraded is assumed to be equal to the degradation rate of Tc in distilled water [43]. The mRNA degradation rate was adjusted such that 20 protein molecules per mRNA transcript are produced [8].

Regarding the affinity of RNAp for the *tetP_A _*promoter, it has been shown to be approximately nine times higher than the combined *tetP_R _*promoters. To facilitate this, the total affinity of the RNAp for the two *tetP_R _*promoters is set equal to 1/9 of the affinity of RNAp for *tetP_A_*. *tetP*_*R*2 _promoter accounts for 95% of the total mRNA while the remaining 5% imputes to *tetP*_*R*1_. Therefore, the binding strength of RNAp to *tetP*_*R*2 _is 19 times higher than the binding strength of RNAp to *tetP*_*R*1_. Moreover, when TetR2 is bound to *tetO2*, the expression of *tetP*_*R*2 _is decreased by 16% and thus, the corresponding kinetic constant is equal to 84% of the case where *tetO2 *is free [32]. Concerning the open complex (between RNAp and DNA) formation kinetic parameter, it is assumed to be equal for all the three promoters.

In general, both the transcription and the translation rates vary significantly. In our formulation, we postulate that the transcription rate is 30 nucleotides whereas the translation rate is 100 codons per second [44].

### Stochastic simulations of systems with chemical reactions

Biological systems are generally not at the thermodynamic limit. They are composed of molecules, such as promoters and operators, that participate in the biological processes in low copy numbers [45-47]. The result is that randomness becomes important. Deterministic approaches cannot take into account this randomness in the behavior of these systems. Stochastic approaches are therefore needed [48,49]. Thus far, several computational methods have been developed that take into account the effects of fluctuations due to the stochasticity [50].

The first attempt to develop an algorithm that allows for discreteness and stochasticity in systems of chemical reactions was made by Gillespie [49]. He developed an algorithm, called Stochastic Simulation Algorithm, that describes accurately the dynamics of a well-mixed system which experiences large fluctuations due to the small number of individual reacting molecules. Although this method can be used specifically to simulate systems with inherent stochasticity, it is inefficient because it simulates each single reaction event separately.

For the purposes of this study, we use a hybrid, stochastic-discrete and stochastic-continuous algorithm to simulate the *tet *operon behavior [26]. Hybrid simulations have been successfully used before to simulate biological systems [5,6,8,25,26,51-54]. The algorithm that we use here is available with open source licenses at http://hysss.sourceforge.net/[25], with a Matlab graphics user interface (GUI), and at http://www.synbioss.org/[30], with an MS Windows GUI. This algorithm divides the system into two subsets. One subset involves the fast reactions that occur in the system. The reactions of this subset are considered continuous Markov processes and the evolution is computed using the Chemical Langenvin Equation. The other subset constitutes the slow reactions of the system. Reactions that belong to this subset are handled as jump Markov processes. In order for a reaction to belong to a subset, two criteria must be satisfied. These criteria are associated with the probabilistic reaction rate (propensity) and the number of molecules that participate in the reaction.

### Underlying assumptions and conditions

The cell is considered to be an isolated, well stirred, homogeneous reactor. It is also considered that the cell volume is equal to 10^-15 ^L and increases exponentially. Cell division is assumed to occur every 30 ± 4 minutes. The exact time for each cell division is randomly chosen from a Gaussian distribution whose mean is set to 30 minutes. Moreover, we hypothesize that during the cell division, the number of molecules of the proteins, mRNAs, and Tc is halved. In order to simplify our model, we do not take into consideration the Tc binding to the Rib assuming that the intracellular Tc amount that is used here is the effective amount that the *tet *operon has to remove from the cell. Furthermore, the diffusion of intracellular Tc out of the cell is considered negligible compared to the Tc removal from the cell by TetA. Finally, the conditions of the cell, such as pH and temperature, are considered to be constant during the simulations.

For each single simulation, 1,000 trials (which correspond to 1,000 cells) were carried out. In what follows, we present not only the average behavior of the 1,000 cells, but also the behavior of single cells. It is important to stress that the use of stochastic algorithms allows for exploring single cell behaviors and variations across the 1,000 cells. This would not be possible with the use of deterministic approaches. The initial conditions that were used in the simulations are 300 Rib, 180 RNAp, 3 promoters (*tetP*_*R*1_,*tetP*_*R*2_,*tetP_A_*), 2 operators (*tetO1, tetO2*), and 4·10^6 ^nsDNA sites. Even though the actual number of Rib and RNAp that exist in the *E. coli *is higher [55], we consider that only a small fraction of the total number is available to this operon.

## Results and Discussion

### Simulation of the tet operon behavior

The first set of simulations explores the steady state of the system. According to experimental observations, at steady state, and in the absence of Tc, there is no intracellular TetA and the amount of intracellular TetR is small. This is a convenient attribute of the *tet *operon. The few TetR proteins require only a small number of Tc molecules to induce the gene expression [56]. Moreover, TetA should not be produced in the absence of Tc because large TetA amounts can lead to cell death [57]. TetA is a membrane protein that pumps Tc out of the cell while pumping protons from the periplasm into the cytoplasm. If TetA is present, even in the absence of Tc, transport of protons through the membrane may result in the collapse of the membrane potential and cell death.

**Figure 2:**
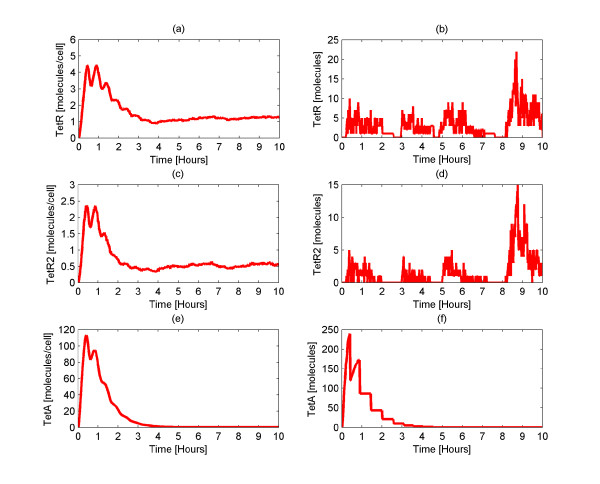
**Amounts of TetR, TetR2 and TetA protein at steady state**. Average (Figures 2a, 2c, 2e) and single cell (Figures 2b, 2d, 2f) number of molecules of TetR, TetR2, and TetA at steady state. Initially, gene expression is allowed to take place. As a result, the amount of the proteins in the cell increases. Once TetR is produced, it represses gene expression allowing the system to reach a steady state at the population level.

In order to investigate the steady state of the system, we conducted a set of simulations in which neither TetA nor TetR initially exist in the cell. Gene expression is therefore allowed to take place producing TetR which inhibits gene expression thus bringing the system to the steady state (at the population level). The results for both the average behavior of the system and the behavior of a single cell are shown in Figure 2. Concerning the average behavior of TetR and TetR2 (Figures 2a, 2c), they reach a maximum of about 5 and 2 molecules per cell respectively and approximately after 3 hours reach steady state. At steady state, there are approximately 2 TetR and 1 TetR2 molecule in the cell. For TetA (Figure 2e), we observe an average initial production of 113 molecules, whereas after 4 hours there is virtually no TetA left in any cell due to dilution. The initial pulse is the result of no initial repression by TetR.

It is important to notice that there is a difference between the average amount of TetA and TetR. This is because the promoter of the *tetA *gene is approximately nine times stronger than the combined *tetR *promoters [19]. This implies that the *tetA *gene expression is very high compared to the *tetR *gene when both of the genes are expressed. Moreover, it should be kept in mind that some of the TetR proteins are bound to nsDNA as well as to Tc, making the amount of free TetR smaller.

Interestingly, looking at the single cell behavior (Figures 2b, 2d) we observe large fluctuations in the number of TetR and TetR2 molecules. According to Figures 2b and 2d, the fluctuations of TetR and TetR2 reach a maximum of 22 and 15 molecules respectively. At steady state, the maximum TetR and TetR2 amount observed across the 1,000 cells is 31 and 38 molecules respectively (data not shown). It is worth mentioning that even though the average number of TetR and TetR2 is small (2 and 1 respectively), there are cells that incorporate TetR2 molecules whose amount fluctuates around high values. However, these cells are only a few and therefore, the average amount of TetR and TetR2 is kept small. Apparently, in this case the average behavior of the cell is not representative of the single cell behavior and this necessitates the use of stochastic algorithms. Had we used a deterministic approach, fluctuations in the behavior of TetR2 could not be established.

Concerning the TetA, in the case of a single cell (Figure 2f), we note that a maximum of 239 molecules is reached. Subsequently, the number of TetA molecules becomes zero after 3.5 hours. At steady state, the maximum number of TetA molecules observed across the 1,000 cells is 348 (data not shown). It should be underlined that cells with high intracellular TetA amounts at steady state cannot survive due to the membrane collapse.

Importantly, these results are consistent with experimental observations, in that not only the number of intracellular TetA molecules is zero at steady state [16,57], but also in that there is only a small amount of free TetR [56]. We have thus established the steady state conditions of TetR and TetA in the cell.

**Figure 3:**
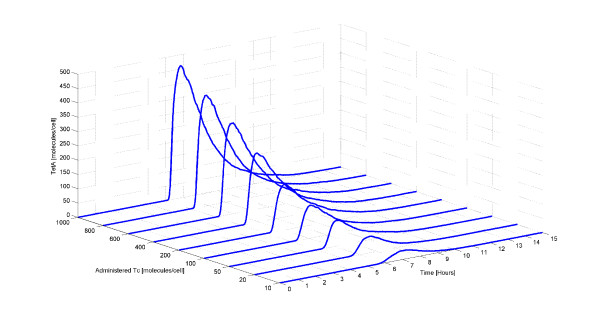
**Average number of TetA molecules for different number of administered Tc molecules**. Initially, there is no TetA in the cell because there is no intracellular Tc. At time equal to 5 hours, a pulse of different Tc amounts is administered to each cell thereby inducing gene expression. Then, the amount of intracellular TetA increases. Increasing amount of administered Tc results in faster production of higher TetA concentration.

Our next step is to test the behavior of the system when a pulse of Tc is administered to the cell. A set of simulations was carried out varying the number of Tc molecules administered to the *E. coli *cells. More specifically, we start with the system at steady state and after 5 hours, a pulse of a wide range of Tc molecules (10,20,50,100,200,400,600,800,1,000) is administered to each cell. Administration of different Tc amounts results in the production of different TetA amounts. The results are portrayed in Figure 3.

Focusing on the TetA amount at time 5 hours, we notice that the larger the quantity of administered Tc, the faster is the response of the system. This is pursuant to the fact that the *tet *operon is a well regulated system which responds fast to the addition of Tc. Furthermore, we observe a nearly linear correlation between the administered Tc and the intracellular TetA amount. An approximately linear dependence between TetA and non-toxic administered Tc amount has been observed before [58]. Given that in our case the administered Tc amount is non-toxic, this result is in agreement with the results by Korpela et al. It is worth stressing that after the removal of Tc from the cell, the system returns again to its original steady state. The time that the system needs to reach the steady state depends on the number of TetA molecules. The higher the number of TetA molecules, the longer it takes for the system to reach the basal state.

Another important fact is that even with the administration of a small Tc amount (10 molecules), the gene expression is turned on automatically in order for the Tc to be removed before causing cell death. This is in line with experimental observations which suggests that induction takes place even with very low, non-toxic Tc concentrations [14,16,18,31,59].

**Figure 4:**
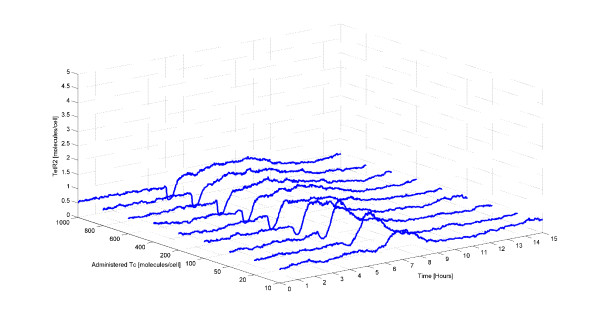
**Average number of TetR2 molecules for different number of administered Tc molecules**. At the beginning, there is about 1 TetR2 molecule in the cell. At time equal to 5 hours, a pulse of various Tc amounts is administered to each cell and thus gene expression is induced. Then, the amount of the free (unbound) TetR2 decreases, because these proteins are occupied by Tc, and thereafter increases upon expression of *tetR*. Increasing amount of administered Tc results in higher decrease in TetR2 amount and longer required time for going back to steady state.

In addition to investigating TetA levels in response to Tc treatment, we also investigate TetR levels at these different Tc concentrations. The average number of intracellular free (unbound) TetR2 molecules practically remains constant regardless of the number of administered Tc molecules. This is an interesting result, especially when contrasted to the TetA behavior. This trend is shown in Figure 4.

As illustrated in Figure 4, the average number of TetR2 molecules decreases slightly when Tc is administered to the cells. This decrease is caused by the binding of Tc to the free TetR2 molecules. However, shortly after the decrease on the TetR2 amount, an increase to the TetR2 amount is observed which comes from the induction of the system which in turn causes the production of many TetR2 molecules. After this increase in the TetR2 amount, the system finally reverts to its steady state. Figure 4 indicates that the higher the amount of administered Tc, the larger the decrease of the TetR2 amount and the longer the time that the system needs to go back to its steady state.

**Figure 5:**
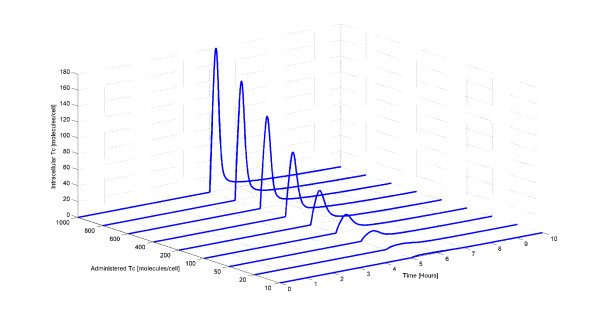
**Average number of intracellular Tc molecules for different number of administered Tc molecules**. Originally, there is no intracellular Tc. At time equal to 5 hours, a pulse of several Tc amounts is administered to each cell thereby inducing gene expression. Then, the amount of free (unbound) intracellular Tc increases and quickly decreases since it binds to TetR2 and is removed by TetA.

The dynamics of the intracellular Tc when different Tc amounts are administered to the cell are also explored. Figure 5 shows the average intracellular Tc amount for the 9 different cases. As expected, the average number of intracellular Tc molecules increases with the number of administered Tc molecules.

**Figure 6:**
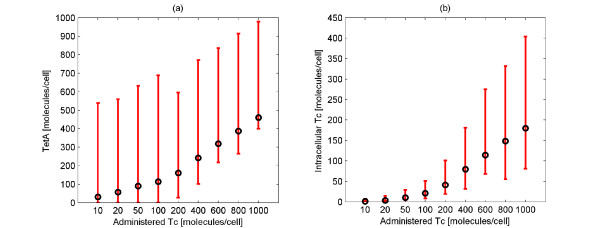
**Maximum average value and variation of the number of intracellular Tc and TetA molecules for different number of administered Tc molecules**. Maximum average value (black color) and variation (minimal and maximal values among the population) (red color) of the number of TetA (Figure 6a) and intracellular Tc (Figure 6b) molecules for different number of administered Tc molecules. The variation of both the number of TetA and intracellular Tc molecules is high.

In Figure 6 we show the maximum value of the mean and the variation (minimal and maximal values among the population) of the number of TetA (Figure 6a,) and intracellular Tc (Figure 6b) molecules for different amounts of administered Tc. As evident in Figure 6a, the maximum TetA amount produced by each cell upon Tc administration varies significantly. It is important to notice that even though the maximum average value of the TetA amount in the cell is non-zero for all the 9 cases, in the first 4 cases (10,20,50,100 administered Tc molecules) there are cells that produce no TetA protein upon Tc administration. These cells would probably not survive from the Tc administration since expression of the resistance protein was not activated and consequently Tc was not removed.

Analyzing Figure 6b, the variation of the number of intracellular Tc molecules seems to be smaller than the variation of the TetA molecules. Furthermore, we note that the higher the number of administered Tc molecules, the higher the variation of the intracellular Tc molecules. It is important to remark that cells whose intracellular Tc amounts lie in the higher regions is less probable to survive while cells with small intracellular Tc amounts is most probable to circumvent the attack of Tc to their transcriptional machinery.

**Figure 7:**
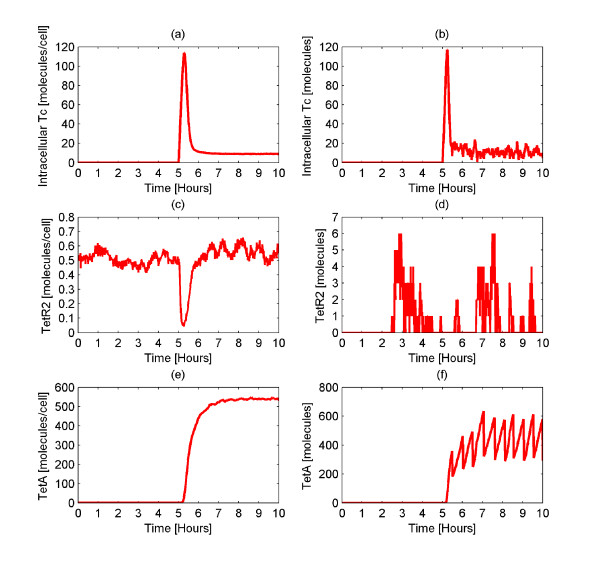
**Amounts of Tc, TetR2 and TetA when 500 Tc molecules are continuously administered**. Average (Figures 7a, 7c, 7e) and single cell (Figures 7b, 7d, 7f) number of molecules of intracellular Tc, TetR2, and TetA when 500 Tc molecules are continuously administered to each cell. Initially, the system is at steady state at the population level. At time equal to 5 hours, 500 Tc molecules are continuously administered to each cell. At steady state, the intracellular TetA amount is high whereas the intracellular TetR2 and Tc amounts are small.

In experimental conditions, *E. coli *usually experience prolonged exposure to Tc. Thus, motivated by the need to understand the behavior of the *tet *operon when in a solution with the antibiotic Tc, we performed a set of simulations where Tc is constantly administered to the cells. The system is initially at steady state. Then, after 5 hours, 500 Tc molecules are administered continuously. This is tantamount to having a constant concentration of external Tc equal to 8.3·10^-11 ^*M *under the following assumptions: 1) the volume of the cells is negligible compared to the volume of the solution, 2) the total Tc amount is ultimately uptaken by the cells, 3) the number of cells in the solution is 10^8^/*mL*. The average behavior of the system (Figures 7a, 7c, 7e) as well as the behavior of a single cell (Figures 7b, 7d, 7f) are illustrated in Figure 7.

Figure 7a shows that when Tc is added to the media, the average intracellular Tc amount reaches a maximum of 114 molecules within the first hour. Subsequently, the system reaches a steady state, at the population level, where there are 9 intracellular Tc molecules. Concerning the average amount of TetR2 (Figure 7c), it decreases almost to the zero value when Tc is administered to the cells. This decrease caused by the binding of Tc to TetR2 which leads to occupation of the free TetR2 molecules. After the induction of the system and consequently the expression of the *tetR *gene, more TetR2 molecules are produced and eventually the average TetR2 amount goes back to its steady state. The average number of TetA molecules (Figure 7e) increases dramatically reaching a maximum of 540 molecules. Afterwards, the system reaches a steady state at the population level where 540 TetA molecules exist in the cell.

Notably, even with a constant high amount of external Tc, the *tet *operon does not allow high levels of internal Tc. This is attributed to the production of high TetA amounts which remove Tc from the cell, thus boosting the performance of the *tet *operon. It should be kept in mind that the intracellular Tc amount is also decreased because of the degradation and the cell division in which the number of Tc molecules is halved every 30 ± 4 minutes. Overall, with constant Tc administration, TetA expression reaches steady state at the population level. At this state, the TetA levels become constant and the intracellular Tc levels are significantly lower than the extracellular ones.

As far as the single cell behavior is concerned, it appears to be similar to the average behavior. Regarding the Tc amount in the single cell (Figure 7b), again after the Tc administration we observe a peak value of approximately 117 molecules. Subsequently, the Tc amount decreases and finally fluctuates around the value of about 9 molecules. Overall, the Tc amount at steady state varies from 0 to 36 molecules among the cells (data not shown). As observed previously (Figure 2), the TetR2 amount of the single cells appears to fluctuate. This amount among the 1,000 cells lies in the area of 0 and 40 molecules (data not shown). Similarly to the average behavior, the single cell behavior with respect to TetA molecules shows an initial increase after Tc administration. This increase results in fluctuations around the value of 500 TetA molecules. At steady state, the TetA amount observed within the single cells varies between 197 and 1,399 molecules (data not shown).

**Figure 8:**
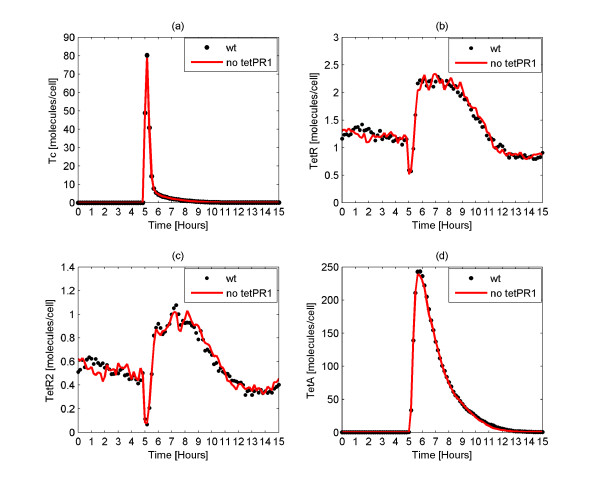
**Comparison of the wild type tet operon with the operon lacking the promoter tetP*_R1_***. Average number of Tc (Figure 8a), TetR (Figure 8b), TetR2 (Figure 8c) and TetA (Figure 8d) molecules of the wild type (wt) *tet *operon as well as of the *tet *operon which lacks the promoter *tetP*_*R*1_. At time of 5 hours, a pulse of 400 Tc molecules is administered to each cell. The behavior of the system appears to be the same with and without the promoter *tetP*_*R*1_.

As discussed previously, only a small portion (approximately 5%) of the total mRNA from the *tetR *gene is transcribed from the *tetP*_*R*1 _promoter [40]. The remaining originates from the *tetP*_*R*2 _promoter. In order to explore the functionality of the *tetP*_*R*1 _promoter, we performed a set of simulations in which no *tetP*_*R*1 _exists in the system. Initially, the system is at steady state and consequently there is neither Tc nor TetA in the cell. At time equal to 5 hours, 400 Tc molecules are administered to the wild type cells as well as to the cells which lack the promoter *tetP*_*R*1_. The trends of the two systems are provided in Figure 8.

Importantly, the results of the simulation of the wild type system are the same with the results of the system which lacks the promoter *tetP*_*R*1_. The number of the intracellular Tc molecules is the same (Figure 8a) in the two systems. In addition, the lack of *tetP*_*R*1 _from the *tet *operon does not appear to affect the amount of the regulatory molecules, TetR, TetR2 and TetA (Figures 8b, 8c, 8d). We further observed that the total behavior of the system is retained even when the *tet *operon includes no *tetP*_*R*1 _promoter (data not shown). This indicates that this promoter is not essential in *E. coli*. It supports the existing hypothesis that this promoter is involved in the *tet *operon because of its functional importance in other bacteria which carry the *tet *operon [40]. This promoter could also have previously played a role in *E. coli*, but it may remain only as a genetic artifact.

### Sensitivity analysis

The *tet *operon is an excellent biological switch [14]. The key features that define its outstanding functionality include high TetA expression in the presence of Tc combined with no appreciable expression leakiness in the absence of Tc. These salient characteristics are attributed to the high affinity of Tc for the repressor TetR2 and the high affinity of TetR2 for the operator sites, *tetO1 *and *tetO2*. Even though these parameters and the associated processes are not rate limiting steps, they do contribute to the fine tuning of this biological switch.

In order to explore the importance of these features, we performed a sensitivity analysis of our model to examine how the different values of the specific *tet *operon parameters influence its behavior. In this set of simulations, a pulse of a wide range (20,50,100,200,400,600, 800,1,000) of Tc molecules is administered to each *E. coli *cell. With this range, we can investigate the sensitivity of the *tet *operon even when minimal amounts of Tc are administered. We can also understand the changes in the behavior of this operon upon increasing the amount of administered Tc.

#### Affinity of Tc for TetR2

First, the importance of Tc's affinity for TetR2 is investigated. As discussed previously, once Tc diffuses into the cell, it binds to TetR2 with high binding strength (*K_eq _*≃ 3·10^9^*M*^-1^) [20]. This causes rapid induction of the system, and consequently the expression of *tetR *and *tetA*. In order to check how this binding strength influences the total behavior of the system, a set of simulations was conducted in which the affinity of Tc for TetR and TetR2 spans two orders of magnitude lower and higher than the nominal value. This could be achieved experimentally by making amino acid substitutions or deletions on TetR2 [60], thereby affecting the affinity of Tc for TetR2. In our analysis, this is realized by changing the kinetic constants that correspond to this affinity (*k*_10_, *k*_12_, *k*_14_, *k*_16_, *k*_20_, *k*_22_) 10 and 100 times.

**Figure 9:**
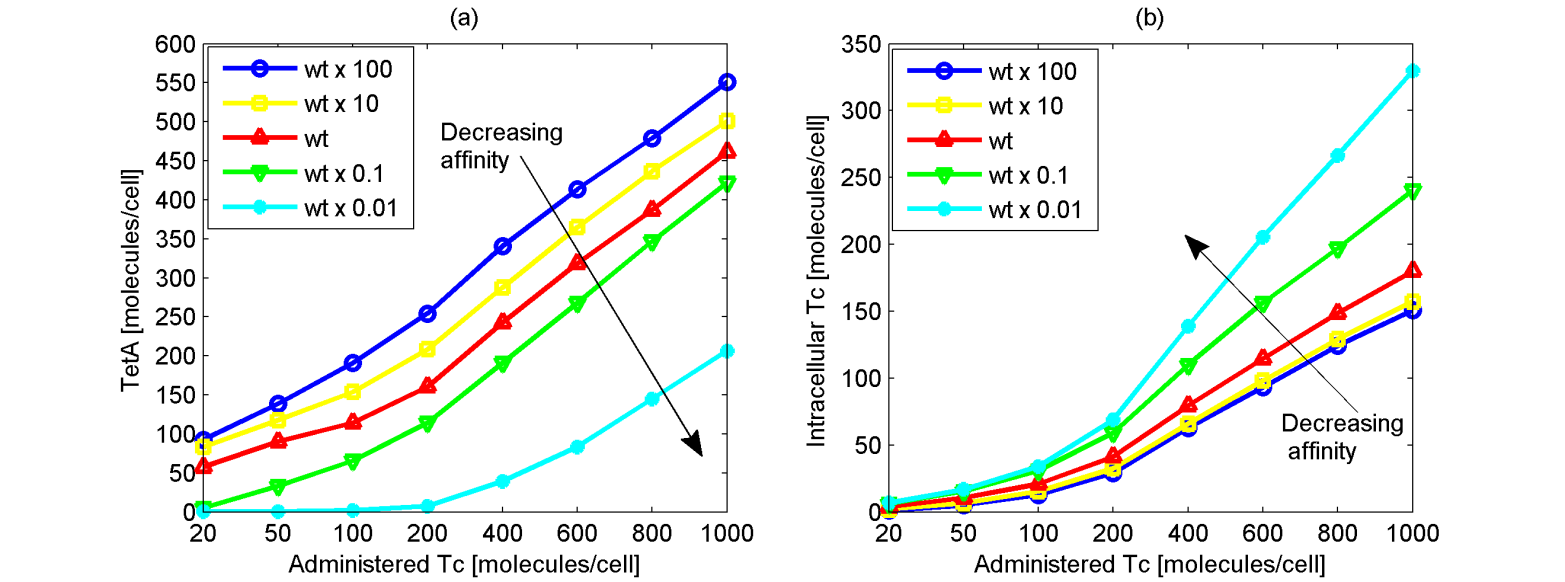
**Impact of changing the affinity of Tc for TetR2 on Tc and TetA amounts**. Average maximum number of TetA (Figure 9a) and Tc (Figure 9b) molecules for a range of administered Tc in the wild type (wt) system, as well as in systems where the affinity of Tc for TetR2 is 10 and 100 times lower and 10 and 100 times higher than the nominal value. Decreasing affinity of Tc for TetR2 results in low TetA and high Tc amounts present in the cell.

Results of the simulations are depicted in Figure 9. They illustrate the maximum value of the average intracellular TetA (Figure 9a) and Tc (Figure 9b) amount when a pulse of various Tc amounts is administered to each cell. The various TetA levels are the result of the range of administered Tc molecules and of different Tc affinities for TetR2.

As depicted in Figure 9, the system is sensitive to changes in the affinity of Tc for TetR2. The average maximum number of TetA molecules that are produced in the presence of Tc increases as the wild type affinity of Tc for TetR2 increases (Figure 9a). As expected, this has an effect on the maximum number of intracellular Tc molecules. This effect is shown in Figure 6b where the average maximum number of intracellular Tc molecules decreases as the wild type affinity of Tc for TetR2 increases. This can be ascribed to the fact that when the affinity of Tc for TetR2 increases, the formation rate of the complex TetR2:Tc2 increases, thereby accelerating gene induction. Therefore, more TetA molecules are generated. As a consequence, Tc is excluded faster from the cell, making intracellular Tc level lower. On the other hand, a decrease in the wild type affinity of Tc for TetR2 leads to a decrease in the intracellular TetA amount (Figure 9a). This decrease in turn elicits a drastic increase on the intracellular Tc amounts (Figure 9b).

It should be kept in mind that the lower the intracellular Tc amount, the higher the probability for the cell to survive. This implies that an increase in the affinity of Tc for TetR2, could help the *E. coli *to survive. On the other hand, a decrease in the affinity of Tc for TetR2 could lead to the faster death of *E. coli*.

Figure 9 indicates that the larger the amount of administered Tc, the larger the differences in the amount of intracellular Tc between the 5 cases (cases with different affinities). Thus, if large Tc amounts are administered, then such changes in the affinity of Tc for TetR2 will cause significantly large differences in the intracellular Tc amount among these 5 cases. Again, this implies that a decrease in the affinity of Tc for TetR2 could result in high intracellular Tc amounts causing cell death.

Another interesting observation is that a large decrease (wt × 0.01) in the affinity of Tc for TetR2, makes the *tet *operon less sensitive to external Tc. In this case, higher Tc amounts must be administered to induce gene expression. In particular, the number of administered Tc molecules must be 200 or higher before expression of *tetA *is turned on (Figure 9a). This is also evident when the affinity is 10 times smaller than the wild type. Then, more than 20 Tc molecules must be administered for *tetA *expression to take place. On the other hand, in the other three cases (wt, wt × 10, wt × 100), gene expression is activated even with the administration of only 20 Tc molecules. This phenomenon is observed because the higher the affinity of Tc for TetR2, the easier and faster the binding of Tc to TetR2, and consequently the gene induction.

For small numbers of administered Tc molecules (20,50,100 molecules), the difference between the 5 cases is not large concerning the maximum average intracellular Tc amount (Figure 9b). However, regarding TetA (Figure 9a), the difference between the 5 cases is large even for small numbers of administered Tc molecules. The high TetA amounts combined with low intracellular Tc amounts could have harmful effects for the cell since high excess of TetA in the cell could lead to cell membrane collapse. Furthermore, when the affinity is larger than the nominal value (wt × 10, wt × 100), little difference in the intracellular Tc amount is noticed. On the other hand, a substantial difference in the intracellular Tc amount is observed in the cases where the affinity is smaller than the nominal value (wt × 0.1, wt × 0.01). This implies that the system is more sensitive to a decrease in the affinity of Tc for TetR2 than to an increase. This probably stems from the fact that the affinity of Tc for TetR2 is already high and not rate limiting, thus higher values do not cause dramatic changes. Furthermore, this indicates that the wild type network affinity is optimal since it is as high as possible to reduce Tc while not increasing TetA significantly.

Lastly, it is worth stressing that our changes in the affinity of Tc for TetR and TetR2 do not affect the average number of TetR and TetR2 molecules when Tc is administered (data not shown). The intracellular amount of these two molecules remains practically the same even upon applying the aforementioned changes.

#### Affinity of TetR2 for the operator sites

**Figure 10:**
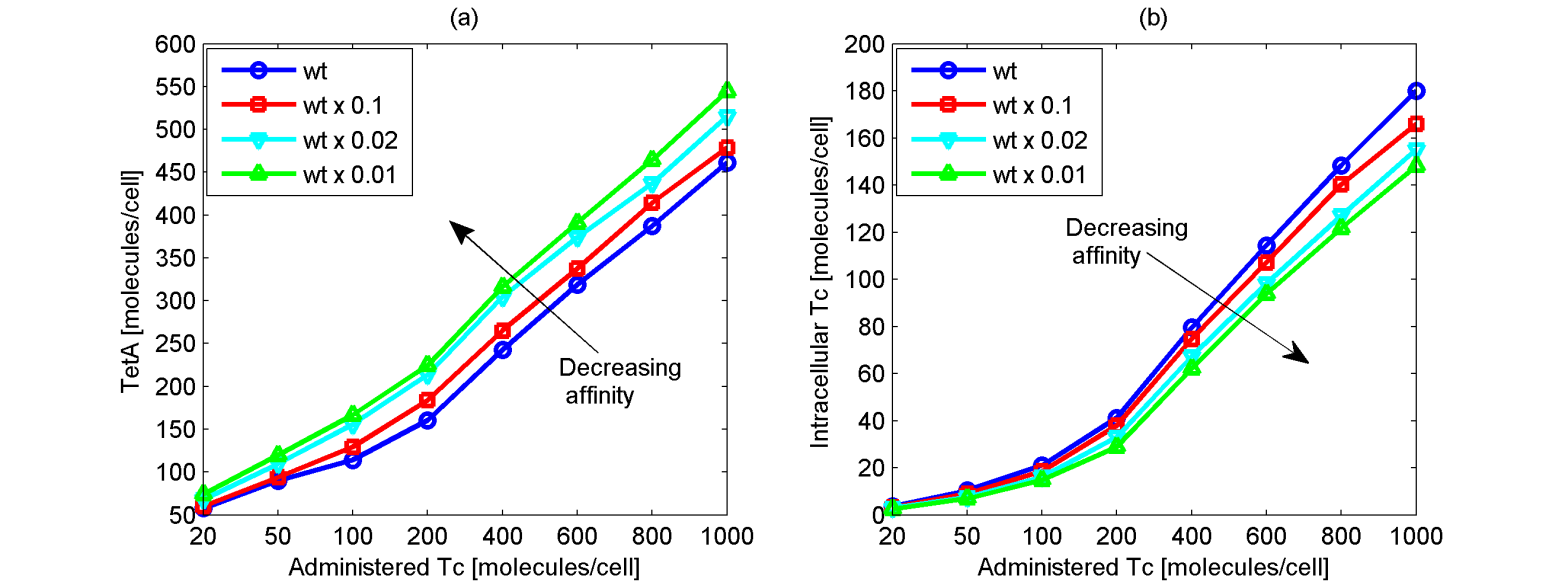
**Impact of changing the affinity of TetR2 for the operators on Tc and TetA amounts**. Average maximum number of TetA (Figure 10a) and Tc (Figure 10b) molecules for a range of administered Tc in the wild type (wt) system, as well as in systems where the affinity of TetR2 for the operators is 10, 50 and 100 times lower than the nominal value. Decreased affinity of TetR2 for the operator sites leads to high TetA and low Tc amounts in the cell.

Here, we explore the significance of the high binding strength (*K_eq _*≃ 10^12^*M*^-1^) [31] of TetR2 to the operator sites, *tetO1 *and *tetO2*. This feature enables the repressor to bind quickly and tightly to the operator sites in the absence of Tc, making this operon a thoroughly tuned biological switch. The importance of this feature is investigated through a set of simulations in which the binding strength of the repressor for the operators is 10, 50 and 100 times lower than the wild type value. This could be experimentally achieved by either introducing amino acid changes on the TetR protein or mutating the operator sites [61]. In our simulations, this is attained by decreasing all the corresponding kinetic parameters (*k*_3_, *k*_5_, *k*_19_, *k*_25_, *k*_64_, *k*_65_, *k*_66_, *k*_67_) 10, 50 and 100 times. We decrease the rate that TetR2 binds to the operators when it is either free (*k*_3_, *k*_5_, *k*_64_, *k*_65_) or bound on Tc (*k*_19_, *k*_25_, *k*_66_, *k*_67_), causing a decrease to the binding strength. In contrast to the previous case, in which the affinity of Tc for the repressor is investigated, we only consider a decrease and not an increase to the binding affinity. The binding affinity value is already very high and a possible increase would not be experimentally meaningful due to diffusion limitations. The results of the simulations are shown in Figure 10 and they represent the average maximum TetA and Tc amounts in the cells.

According to the results, the fidelity of the *tet *operon appears to be susceptible to changes in the affinity of TetR2 for the operators. A decrease in the affinity of TetR2 for the operators results in an increase in the amount of generated TetA (Figure 10a). Thus, lower affinities give rise to higher TetA production. Higher TetA amount leads to faster Tc removal from the cell thereby leading to lower intracellular Tc amounts (Figure 10b). The decreased amount of intracellular Tc is beneficial for the cell. However, the decrease in the affinity of TetR2 for the operators results also in an increase in the TetA, TetR and TetR2 amount at steady state. This causes the *tet *operon to loose its unique function. In other words, the trade off in having Tc removed fast from the cell is the high intracellular TetA, TetR and TetR2 amounts at steady state.

As remarked previously, *tet *operon is a unique biological switch [14]. This is attributed to the optimal design of high affinity levels which accommodates 1) small number of TetR2 molecules at steady state, which allows gene induction to occur with minimal Tc amounts, and 2) tight repression of *tetA *gene, which does not allow the production of TetA in the absence of Tc. Therefore, at steady state, the intracellular TetR and TetR2 amount is small (with average values 2 and 1 molecules respectively) and the TetA amount is zero. However, here we observe that when the affinity of TetR2 for the operator sites decreases, the amount of TetA, TetR and TetR2 increases. In particular, when the affinity is 10, 50 and 100 times lower than the wild type value, the number of intracellular TetA molecules at steady state is 3, 7, and 10 respectively (data not shown). The lower the affinity of TetR2 for the operator sites, the higher the TetA amount at steady state. Thus, the decrease in the affinity of TetR2 for the operators results in non-zero TetA amounts at steady state. This in turn could cause cell death because TetA, in the absence of Tc, is toxic for the cell [57].

Additionally, when the affinity of TetR2 for the operators is 10, 50 and 100 times lower than the nominal value, the average number of intracellular TetR and TetR2 molecules is 3, 4, 5 and 2, 3, 5 respectively (data not shown). Given that the TetR and TetR2 amount in each single cell experiences large fluctuations, the higher the mean value, the higher the fluctuations in the TetR and TetR2 amount. Furthermore, the higher the intracellular TetR and TetR2 amount, the higher the required Tc amount for inducing gene expression. This happens because the administered Tc is occupied by the free TetR and TetR2 molecules and therefore cannot bind to the TetR2 bound on the operators to activate expression. Therefore, the large intracellular TetR and TetR2 amounts require large Tc amounts to induce gene expression thereby eliminating the functionality of the *tet *operon. Thus, the aforestated changes in the amount of TetR, TetR2 and TetA at steady state are detrimental to the *E. coli*. This confirms that *tet *operon is a very well tuned system and possible changes in its crucial parameters could be harmful for the *E. coli*.

It is noticeable that the differences in Tc and TetA amounts that are caused by changing the affinity of Tc for TetR2 are larger than the differences that are caused by modifying the affinity of TetR2 for the operator sites. This indicates that the *tet *operon is more sensitive to changes in the affinity of Tc for TetR2 than in the affinity of TetR2 for the operators.

## Conclusions

We have developed a detailed mathematical model for the *tet *operon and performed stochastic simulations to examine the mechanisms that govern the dynamics of this interesting biological system. Conducting stochastic simulations, we investigated the average behavior of 1,000 cells, the variability across the cells, and finally the single cell behavior. The results of the simulations are in agreement with, and explain well numerous experimental observations such as tight repression, fast gene expression, induction with small Tc amounts, and small intracellular TetR2 amounts.

Our simulations demonstrate that there is a nearly linear relationship between the administered Tc and the TetA amount. Furthermore, the results indicate large fluctuations in the amount of the repressor protein TetR2. Additionally, our findings highlight that the behavior of the *tet *operon is the same even when it lacks the promoter *tetP*_*R*1_^. ^This could imply that this promoter is redundant and not necessary in *E. coli*, although it may be functionally important in other bacteria. It could also indicate that although *E. coli *used to need this promoter, they do not need it anymore and it just exists in their genome.

Sensitivity analysis illustrates that the affinity of Tc for the repressor TetR2 and the affinity of TetR2 for the operator sites have a high impact on the behavior of the *tet *operon, suggesting optimum interaction strengths developed through natural selection. In particular, an increase in the affinity of Tc for the repressor leads to an increase in the production of TetA protein. Increased TetA amounts remove Tc from the cell faster, thereby keeping the levels of the intracellular Tc low. A decrease in the affinity of the TetR2 for the operators results in the production of more TetA protein upon Tc administration. Additionally, it results in an increase in the number of TetR, TetR2 and TetA molecules at steady state. This causes a decrease in the number of intracellular Tc molecules, increasing the probability for the cell to survive. However, the existence of intracellular TetA at steady state may lead to cell death.

The need for a mechanistic understanding of bacterial resistance to Tc was identified many years ago [59,62]. Computer simulations of the *tet *operon provide a comprehensive understanding of the interactions between the *tet *operon molecular elements. They also provide valuable information that may contribute to the design of prototype synthetic gene regulatory networks.

## Authors' contributions

KB developed the model, carried out the simulations and wrote the manuscript. PD participated in the design of the study and helped to write the manuscript. YK conceived of the study, and participated in its design and coordination and helped to write the manuscript. All authors read and approved the final manuscript.
